# Health effects associated with measured levels of contaminants in the Arctic

**DOI:** 10.3402/ijch.v75.33805

**Published:** 2016-12-13

**Authors:** Pál Weihe, Fróði Debes, Jónrit Halling, Maria Skaalum Petersen, Gina Muckle, Jon Øyvind Odland, Alexey Dudarev, Pierre Ayotte, Éric Dewailly, Philippe Grandjean, Eva Bonefeld-Jørgensen

**Affiliations:** 1 Department of Occupational Medicine and Public Health, The Faroese Hospital System, Torshavn, Faroe Islands; 2 École de psychologie, Université Laval and Centre de recherche du CHU de Québec, Québec, City, QC, Canada; 3 Department of Community Medicine, UiT The Arctic University of Norway, Tromso, Norway; 4 Northwest Public Health Research Center, St. Petersburg, Russia; 5 INSPQ, Université de Laval, Québec City, QC, Canada; 6 Department of Environmental Medicine, Univerisity of Southern Denmark, Odense, Denmark; 7 Centre for Arctic Health, Department of Public Health, Aarhus University, Aarhus, Denmark

**Keywords:** effects, contaminants, Arctic, neurobehavioural, immunological, reproductive, cardiovascular, endocrine, carcinogenic

## Abstract

The Human Health Assessment Group has over the past decade recommended that effect studies be conducted in the circumpolar area. Such studies examine the association between contaminant exposure in the Arctic populations and health effects. Because foetuses and young children are the most vulnerable, effect studies are often prospective child cohort studies. The emphasis in this article is on a description of the effects associated with contaminant exposure in the Arctic. The main topics addressed are neurobehavioural, immunological, reproductive, cardiovascular, endocrine and carcinogenic effect. For each topic, the association between exposure and effects is described, and some results are reported for similar studies outside the Arctic.

## Neurobehavioural effects

### Mercury

Follow-ups of the first Faroese cohort, established from 1,022 consecutive singleton births in 1986 and 1987, have demonstrated permanent negative neurobehavioural effects of foetal exposure to Hg, from the pregnant women's consumption of whale meat, even at low levels. As the findings are among the most important at present, they are summarized in some detail.

At age 7 years, clinical examination and neurophysiological testing of 921 children did not reveal any clear-cut Hg-related abnormalities. However, neuropsychological dysfunctions were observed in language, attention and memory, and to a lesser extent in visuospatial and motor functions. These associations remained after adjusting for covariates and after excluding children whose mothers had hair MeHg concentrations over 10 µg/g (50 nmol/g). Thus, the effects were widespread and detectable at exposure levels currently considered safe (1).

In examinations of 878 children at age 14 years (2), prenatal MeHg exposure was significantly associated with deficits in finger-tapping speed, in reaction time on a continued performance task and in confrontation naming. Post-natal exposure had no discernible effect. These findings are similar to those obtained at age 7 years, and the relative contribution of MeHg exposure to the predictive power of the multiple regression models was also similar, and MeHg-associated test score differences had not changed between the two examinations. In structural equation model analyses with five latent variables, MeHg exposure was significantly associated with deficits in motor, attention and verbal functions. These findings were supported by independent assessment of neurophysiological outcomes. Thus, the effects on brain function appear to be multifocal and lasting.

In brainstem auditory-evoked potential (BAEP) latencies (3), latencies of peaks III and V increased by about 0.012 ms when the cord blood MeHg concentration doubled. As seen at age 7 years, this effect appeared mainly within the I–III interpeak interval. Despite lower post-natal exposures, hair Hg level at age 14 years was associated with prolonged III–V interpeak latencies. All benchmark dose results were similar to those obtained for dose–response relationships at age 7 years. Thus, the persistence of prolonged I–III interpeak intervals indicates that some neurotoxic effects from prenatal MeHg exposure are irreversible. A change in vulnerability to MeHg toxicity is suggested by the apparent sensitivity of the peak III–V component to recent MeHg exposure (3).

Prenatal MeHg exposure was associated with decreased sympathetic and parasympathetic modulation of the heart rate variability. Parallel MeHg-related delays of BAEP latencies may be caused by underlying MeHg neurotoxicity to brainstem nuclei (4).

At age 22 years, 830 of the cohort members were re-examined and administered an extended neuropsychological test battery, covering eight broad ability domains. Effects of MeHg exposure on single neuropsychological outcomes were tested in multiple regression analyses after correction for the same obligatory covariate model as applied in the 14-year-old study. Of the single test variables, six were adversely affected by MeHg to a statistically significant degree after correction for the covariate model: Boston Naming Test, with and without cues; Synonyms, Woodcock-Johnson III (WJ III); Antonyms, WJ III; Block Design, Wechsler Adult Intelligence Scale-Revised, last 3 items; California Verbal Learning Test, Trial 1 (5). The vast majority of the variables were affected in a negative direction, and for each broad ability domain the balance was also in a negative direction, likely reflecting a weak negative effect in the data set.

In a brief measurement model, a latent variable for general intelligence was defined, affecting two subordinate latent variables, fluid intelligence and crystallized intelligence, with two and five manifest indicator variables, respectively, all corrected for a preselected set of 11 covariates. Another measurement model was defined for a latent MeHg exposure variable with three logarithmically transformed manifest indicator variables. A structural model was then specified with the latent exposure variable affecting the latent variable for general intelligence (5). The model fit was acceptable to good. The standardized effect of the latent exposure variable on the latent variable for general intelligence was −0.145 and was significant (p=0.002). Transformed to the intelligence quotient (IQ)-scale, with mean 100 and standard deviation 15, this signifies a loss of 2.2 IQ-points for a 10-fold increase in MeHg exposure.

An extended measurement model was subsequently defined with general intelligence broadly specified to affect as many as seven subordinate ability domains (Verbal comprehension, Visuo-Spatial Processing, Short-Term Memory, Long-Term Storage and Retrieval, Cognitive Processing Speed, Timed Reaction and Decision Speed, Psychomotor Speed, and Dexterity), with between two and seven manifest indicator variables each. This model also fitted the data well. The effect on general intelligence was −0.093 and significant (p=0.041). This corresponds to a loss of 1.4 IQ-points for a 10-fold increase in MeHg exposure.

In a model for the seven broad ability domains alone, all were negatively affected by MeHg, with the effect on crystallized intelligence being highly significant.

These results have wide implications, because domain-independent general mental ability enters into all specific and differential abilities and is recognized as the strongest single-most predictor in the social sciences for success in education and occupation, as well as in many other areas of life. It also decreases the possibilities for neural or behavioural compensation as late as age 22 years, thereby substantiating a lasting impairment of the intellect and an adverse impact on the future life outcomes of the exposed individuals.

This was demonstrated in recent analyses which found that as latent educational attainment at 16 years is significantly dependent on general cognitive ability at 7 years which again is significantly negatively affected by prenatal MeHg, a highly significant mediated (or indirect) negative effect of prenatal MeHg on educational attainment at 16 years can be seen (6). No direct effect or total effect was found. The same indirect effects were observed at the level of the individual school subjects. Still for Danish spelling, a direct and a total negative effect were observed. Similarly, a highly significant indirect negative effect of prenatal MeHg was seen on the educational status reached at age 22 years (high school graduation or start of advanced studies).

In the mixed composition of determinants of educational attainment (7,8), some are significantly negatively affected by MeHg (cognitive factors), whereas others may be less so (factors of personality and mental health), thereby attenuating direct and total effects of prenatal MeHg on educational attainment and status.

In the Nunavik Child Development Study (NCDS), a prospective mother–child cohort study taking place in Nunavik (Arctic Quebec), results obtained through both neurobehavioural and electrophysiological testing with children aged 11 years suggested that prenatal Hg exposure is associated with poorer perceptual processing, attentional mechanisms, memory and intellectual function (9–13).

Behavioural assessments of 11-year-old children were obtained from two questionnaires completed by their classroom teacher providing scores of attention, internalizing or externalizing problems and four clinical diagnoses. Cord blood MeHg concentrations were significantly related to attention problems (14), and they showed for the first time that prenatal MeHg exposure constitutes a risk factor for attention deficit hyperactivity disorder (ADHD) likely to interfere with learning and performance in the classroom. Likewise, cross-sectional evidence links MeHg exposure to autism spectrum disorder (15). However, the evidence available is limited and so conclusions regarding autism or ADHD must be drawn with caution.

Negative effects on the visual system from exposure to MeHg have been shown in an adult-exposed population in the Amazon. None of the environmental contaminants considered in the NCDS were associated with negative effects on visual acuity, colour perception or contrast sensitivity, but a significant association between cord blood MeHg and event-related potential (ERP) amplitude at the highest contrast level suggests deficits in the parvocellular system, which is specialized in high-contrast vision, visual acuity and colour vision. This is supported by deficits in acuity and colour vision reported in association with prenatal Hg exposure in other studies (16–18). A lack of significant neurotoxic effects on visual evoked potentials (VEP) in school-age children from Greenland (19) and in 7-year-old Faroese children (1,20) may have been due to differences in testing protocols. By manipulating visual contrast levels, the NCDS protocol was designed to optimally detect subtle effects. Changes in VEP latency in association with cord Hg concentrations were previously reported in preschoolers from Nunavik (21). NCDS results at age 11 years show that this negative effect on latency persists at school age.

The MeHg effects seen in the NCDS corroborate those reported in the Faroe Islands and New Zealand. Results from the higher exposed Faroese and Nunavik cohorts are also observed in studies conducted in lower Hg-exposed populations like the Project Viva study in Boston, where fish consumption is higher than average for the United States. Here, mean maternal hair Hg concentration of 0.55 µg/g (22) was associated with a reduction in children's cognition at 6 months of age and again at age 3 years. Comparable results were obtained in New York City at similar exposure levels (23).

The available evidence suggests that cognitive impairment occurs at MeHg exposure levels prevalent in general populations elsewhere and is a matter of public health concern. Since 2000, prevention efforts have relied on the recommendations of the US National Research Council to maintain MeHg exposure below a reference dose of 0.1 µg/kg body weight per day (24). However, prudent advice is to minimize exposure to the extent possible, because a threshold for adverse effects on brain development may not exist (25).

Adverse effects of MeHg and beneficial effects of seafood nutrients on neurodevelopment may mask each other (26). Studies in Boston and New York City both showed that benefits from the mother's seafood diet to the child's brain development were less when Hg exposure was higher. Data from the NCDS showed that the prenatal Hg effect on intellectual function became stronger when cord docosahexaenoic acid was also considered (13). Data from the Seychelles show that cognitive development in children is associated with neither maternal fish intake nor MeHg exposure, when examined one at a time. Only if maternal fish intake and Hg are accounted for simultaneously fish intake is clearly beneficial, while Hg has negative effects (27). Thus, the positive and negative effects appeared to offset one another.

Post-natal exposure to MeHg is also likely to cause adverse effects on the continued development of the nervous system. Inconsistent evidence may be due to difficulties in characterizing the trajectory of post-natal exposure. Neurophysiological assessment shows that post-natal exposure up to the teenage years can cause harm (3). Thus, both pregnant women and children should be considered populations at increased risk (28).

Effects associated with MeHg exposure have been documented in humans at successively lower exposures as a result of better study designs, larger groups of subjects, more sensitive methodology and better control of confounding factors. It is likely that future studies will continue to identify effects at lower exposures than those considered safe today.

### Lead

In the first Faroese birth cohort, the effect of prenatal lead (Pb) exposure in the presence of similar molar level in cord blood to MeHg was evaluated. A total of 896 cohort subjects participated in a clinical examination at age 7 years and 808 subjects in a follow-up at age 14 years. The association between cord-blood Pb concentration and cognitive deficits (attention or working memory, language, visuospatial, memory) was evaluated using multiple regression models. After including statistical interaction terms, Pb-associated adverse effects on cognitive function were observed in subjects with a low MeHg exposure. In particular, higher cord-blood Pb was associated with a lower digit span forward score on the Wechsler Intelligence Scale for Children-Revised (β=−1.70, 95% confidence interval (CI) −3.12 to −0.28) at age 7 years and a lower digit span backward score (β=−2.73, 95% CI −4.32 to −1.14) at age 14 years. Some interaction terms between Pb and MeHg suggested that the combined effect of the exposures was less than additive (29).

In the NCDS, results obtained with 5- and 11-year-old children through both neurobehavioural and ERP testing suggested that prenatal Pb is related to poorer cognitive development and intellectual function (9,13). Furthermore, 11-year-old blood Pb concentrations were associated with externalizing problems (14). NCDS results relating to post-natal Pb exposure and child behaviour replicate those of several previous studies (reviewed by Eubig et al. (30)), although the main source of Pb exposure in Nunavik, lead shot, is unique in the Pb exposure literature.

### Persistent organic pollutants

Grandjean et al. (31) analysed banked cord blood from the first Faroese birth cohort to determine the possible neurotoxic impact of prenatal exposure to polychlorinated biphenyls (PCBs). A total of 917 members completed a series of neuropsychological tests at age 7 years. Major PCB congeners (PCB118, PCB138, PCB153 and PCB180), the calculated total PCB concentration and the PCB exposure estimated in a structural equation model showed weak associations with test deficits, with statistically significant negative associations only with the Boston Naming Test. Likewise, neither hexachlorobenzene (HCB) nor *p,p’*-dichlorodiphenyldichloroethylene (*p,p’*-DDE) showed clear links with neurobehavioural deficits. In a structural equation model with motor and verbally mediated latent variables, the PCB effects remained weak and virtually disappeared after adjusting for MeHg exposure, whereas Hg remained statistically significant. Thus, in the presence of elevated MeHg exposure, PCB neurotoxicity may be difficult to detect, and PCB exposure does not explain the MeHg neurotoxicity previously reported in this cohort (31).

Traditional and electrophysiological testing in the NCDS found negative effects of prenatal PCB exposure on child cognitive development in participants that had been breastfed for a short period. In addition, post-natal PCB exposure affects processes associated with error monitoring (11,12). However, the NCDS failed to confirm the adverse effects of prenatal PCB exposure on IQ reported in Michigan (32) and Oswego (33) but comparison of the congener profile in Nunavik with that in the Michigan cohort suggests that the PCB mixture to which the children were exposed was likely to be less neurotoxic than in the Michigan study.

## Immunological effects

Certain environmental pollutants can adversely affect the development of the immune system (34–41).

The high incidence of infectious diseases – particularly meningitis, bronchopulmonary infections and middle ear infections – in young children from Nunavik has been known for many years (42). In view of the immunotoxic properties displayed by some organochlorines (OCs), in particular following perinatal exposure, it has been hypothesized that part of the high infection incidence among Inuit infants could be related to the relatively high maternal body burden of these contaminants and their partial transfer to newborns during breastfeeding. To test this hypothesis, three epidemiological studies have been conducted during the past 20 years in Arctic Quebec to investigate the relationship between pre- or post-natal OC exposure, immune status and the occurrence of infectious diseases among Inuit infants. Results in three different groups of Inuit children indicated that prenatal exposure to OCs increases susceptibility to infectious diseases, and in particular to otitis media (38,43,44).

The Faroese studies provide epidemiological data on human immunotoxicity – as reflected by a reduction in serum antibody production after routine childhood immunizations – in relation to developmental exposures to environmental chemicals (45–47). The studies showed that developmental and perinatal exposure to PCBs and Perfluorinated Chemicals (PFCs) from marine food and other sources may inhibit immune function, as indicated by deficient serum concentrations of antibodies against childhood vaccines. Results from the Faroe Islands show that the risk of having an antibody concentration below 0.1 IU/mL at age 7 years increased at higher levels of exposure to PCBs and PFCs. The results suggest that PFCs have an even stronger negative effect than PCBs on serum antibody concentrations (45–47). For PCBs, a doubling of the serum concentration at age 18 months was associated with a decline of 20% in the antibody level at age 7 years. After the completion of breastfeeding and associated transfer of PCBs, the child at age 18 months has an average serum PCB concentration similar to that of the mother (46), after which the concentration declines as the body lipid compartment continues to expand. For PFCs, the recent accumulation was found to be the most important predictor of immunotoxicity: A doubling in serum PFC concentration measured at age 5 years was linked to a decrease of up to 50% in the antibody concentration at age 7 years (p<0.001). Due to the long half-life of PFCs (48), serum concentrations at early school age are expected to be relatively stable.

## Reproductive effects

In 1992, Carlsen and co-workers published a combined analysis of results from 61 papers published between 1939 and 1991 and showed a significant decline in sperm count over the 50-year period. A detailed reanalysis of the results found that their conclusion was supported by the underlying studies (49,50). Following the 1992 publication, many researchers retrospectively analysed their historical data for temporal trends, some finding a decline and others not.

The causes of decreased semen quality are not clear, but it is feasible that many cases may have been caused by exposure to environmental factors in utero, during adolescence or in adulthood (51), probably also acting against a backdrop of different genetic susceptibility to environmental exposure.

The median sperm concentration of fertile men in a semen quality study conducted in Greenland in 2004 was 53 million/mL, with a median sperm cell volume of 3.2 mL, a total sperm count of 186 million and a median motility of 60% (52). No regional difference was found in sperm count, but sperm cell motility differed among regions. In a following study, Toft et al. (52) found that sperm concentration was not impaired by increasing serum PCB153 or *p,p’*-DDE levels in Greenlanders. Also, that there was no association between the proportion of morphologically normal sperm and either PCB153 or *p,p’*-DDE concentration in blood. However, sperm motility was inversely related to PCB153 concentration in this population.

Results concerning male reproductive toxicity in the CLEAR study (see AMAP Assessment 2015: Human Health in the Arctic, Chapter 2) indicated that exposure to perfluorooctanesulfonic acid (PFOS) was associated with more abnormal sperm morphology (53) but that PFCs were not consistently associated with other markers of male reproductive function, including reproductive hormones and markers of sperm DNA damage (54). There was no observed change in male reproductive function at higher levels of polybrominated diphenyl ethers (PBDEs) and Hg exposure (55,56). However, menstrual cycle characteristics were adversely affected at higher levels of exposure to PFCs as indicated by longer menstrual cycles in women in the highest tertile of PFOS exposure compared to the lowest (57).

In a recent study on testicular function in the Faroe Islands, Halling et al. (58) found lower sperm concentrations for Faroese men than for Danish men (crude median 40 million/mL vs. 48 million/mL, p<0.0005). However, because semen volume was higher in the Faroese men, the total sperm counts did not differ (159 million vs. 151 million, p=0.2). Similarly, there was no overall difference between the two populations in terms of sperm motility or morphology. Recent data have shown sperm count to be low in young men from several European countries, but slightly higher than among the Danes (59–61). This indicates that semen quality for both Danish and Faroese men seems to be low compared to men from other European countries.

The inhibin B:follicle-stimulating hormone (FSH) ratios for the Faroese men were lower than for the Danes (64 vs. 76, p=0.001). Similarly, a lower total testosterone:luteinizing hormone ratio (T:LH; 4.6 vs. 6.0, p<0.0005) and a lower calculated free-testosterone:luteinizing hormone ratio (FT:LH; 94 vs. 134, p<0.0005) were detected for the Faroese men (58). The low inhibin B:FSH ratio for the Faroese men corroborates the finding of low sperm count and provides independent evidence of poorer testicular function in the Faroese men than in the Danes, although the medians were at a level where the association between sperm count and inhibin B is weakened (62). The lower T:LH and FT:LH ratios indicate a lower Leydig cell capacity among Faroese men compared to Danes. Thus, the level of total testicular function among Faroese men may be the same or lower than for the Danes.

The reason for low testicular function in the Faroese young men is unclear, but could be due to high exposure to persistent organic pollutants (POPs). Studies have shown associations between high PCB levels and low semen quality, and because PCBs and *p,p’*-DDE have the potential to interfere with sex hormone function (63,64), it could be assumed that these compounds can affect the function of the hormone-producing organs (65). Some reports on the effect of POPs on male reproduction in humans indicate weak negative effects on sperm motility (65–67). Among the Faroese men, this study found the percentage of motile cells to be significantly lower compared to Danish men, indicating that increased exposure to endocrine disruptors may be one explanation for the difference.

Serum steroid hormone-binding globulin (SHBG) levels for the Faroese men were much higher than for the Danes. One explanation could be the high PCB levels among the Faroese. Grandjean et al. (68) reported that SHBG increased at higher PCB exposure, both prenatally and post-natally. Because PCBs are known to affect a number of liver functions, it may be that PCB-induced hepatic SHBG synthesis could play a role, although this remains to be confirmed (68).

Contaminant effects have also been observed on foetal growth and growth during childhood. In the NCDS, weight, height and head circumference were measured at birth and during childhood. Path analyses were conducted to model the longitudinal relations between exposure variables and growth outcomes in newborns and children. Detailed results were presented by Dallaire et al. (69). Prenatal exposure to PCB153 and Pb was not associated with foetal growth. However, prenatal exposure to Pb, but not childhood Pb exposure, was related to shorter height in childhood. Plasma PCB levels in 11-year-olds were moderately related to smaller height, weight (controlled for height), head circumference and body mass index (BMI) at school age. In the sample of children followed at 11 years of age, in utero exposure to PCB153 was not related to foetal growth, but in another sample from the same population (n=248 pregnant women) cord PCB153 and Hg concentrations were related to shorter duration of pregnancy, a recognized determinant of foetal growth (69), and their associations with reduced foetal growth were mediated through their relation with a shorter gestation duration. PCBs are present in the environment as complex mixtures of different congeners, and the relative proportions of the congeners that comprise these mixtures can differ markedly between various geographic regions. Failure to detect direct effects on foetal growth, as observed in studies in Europe and elsewhere, suggests that the congeners forming the PCB mixture found in the Arctic might be less toxic. The NCDS results support findings from two other studies on children moderately exposed to PCBs, indicating that chronic exposure to PCBs during childhood can adversely affect skeletal growth and body weight (70,71). Consistent with results from the NCDS, cord blood Pb concentrations are not related to foetal growth in most studies (72–76), with one exception (76). The NCDS is the first study providing empirical evidence that prenatal Pb exposure is related to poorer growth in school-age children.

## Cardiovascular effects

### Mercury

Possible cardiovascular effects of Hg have recently emerged in the scientific literature (77). A growing body of evidence suggests that MeHg exposure can increase risk of adverse cardiovascular impacts in exposed populations. The link between MeHg and acute myocardial infarction or sudden cardiac death is still debated in low Hg exposed populations (78,79).

Contradictory results have been reported on Hg exposure and the risk of hypertension (80). In Nunavik adults, a retrospective analysis of the 1992 survey reported no association between Hg and high blood pressure (81). Based on the 2004 data, however, Hg was associated with increased blood pressure and pulse pressure (82,83). In the Faroe Islands, high blood pressure was found to be associated with Hg exposure among male whale hunters (84). In Greenland, no association was found between Hg exposure and high blood pressure (85). Associations between Hg exposure and blood pressure were also studied in children. Associations were reported between prenatal Hg exposure and lower systolic blood pressure in 7-year-old Faroese children (86) and for lower diastolic blood pressure in the Seychelles (87). In Nunavik children, no associations were found between blood pressure and either cord blood or contemporary Hg exposure at age 11 years (88).

Heart rate variability has also been studied in Arctic populations. An association was reported between Hg exposure and decreased heart rate variability in adults from Nunavik (82). Similar results were reported among James Bay Cree adults (89). In children from Nunavik, cord blood Hg concentrations were not related to heart rate variability parameters at age 11 years, but child blood Hg levels were associated with decreased overall heart rate variability parameters, and these associations remained significant after adjusting for cord blood Hg, n-3 polyunsaturated fatty acids (PUFA) and selenium. In Faroese children, cord blood Hg concentrations were related to reduced low-frequency (LF) activities at age 7 years as well as with reduced LF, high frequency (HF), HF variation and coefficient of variation for the R–R interval of the electrocardiogram at age 14 years, and hair Hg at age 7 years was associated with LF and LF variation coefficient (4). A difference that is likely to explain discrepancies between findings with regard to cardiac autonomic activity in childhood is the consideration of cord n-3 PUFA and selenium in Nunavik: A significant negative association between cord blood Hg and NN (standard β=−0.13, p=0.05) was observed after adjusting for most of the traditional risk factors used in the Faroe Islands studies (age, sex, birthweight, child BMI and smoking during pregnancy), but these associations were no longer significant after adjusting for cord n-3 PUFA and selenium. This indicates that not adjusting for the nutrients found in abundance in fish could overestimate the prenatal Hg effect. Differences in study findings might also be attributable to differences in Hg exposure between cohorts. In fact, average cord blood Hg was about 1.5-fold higher among Faroese children (90) than those from Nunavik, and hair Hg at age 7 years was three times higher in the Faroese study than the Nunavik study. Prenatal Hg exposure was also higher in the Seychelles study than in Nunavik (87).

The predictive value of heart rate variability parameters in healthy children and risk of chronic diseases are unknown. Nevertheless, results from the Faroese and Nunavik cohorts provide evidence that Hg exposure during childhood is related to changes in cardiac autonomic activity at school age.

## Endocrine effects

Endocrine-disrupting chemicals (EDCs) interfere with the endocrine system and can result in adverse developmental, reproductive, neurological, cardiovascular, metabolic and immune effects. An EDC is defined as “an exogenous substance or mixture, that alters the function(s) of the endocrine system, and consequently causes adverse health effects in an intact organism or its progeny or (sub)-population” (91).

## Biomarkers of POPs exposure and their hormone-disrupting effects

Since 2000, parallel studies have been undertaken in Greenland on the human monitoring of biomarkers for POPs exposure and biomarkers of POPs effects, focusing on hormone-disruptive potentials and genetic sensitivity biomarkers (63,64). In Greenland, regional differences and sex differences (highest in men) are observed in serum POP levels. The highest levels are found in Inuit living on the east coast (Ittoqqortoormiit and Tasiilaq) and in the north-west (Qaanaaq) (92–94).

Today, it is well known that the levels and profiles for the various POP groups vary among Greenlandic districts (64). Studies on biomarkers of toxicological effects have shown that individual POPs have very different biological potentials. For example, some PCB congeners possess an estrogenic potential (e.g. some hydroxy-PCBs), whereas others are antiestrogenic (e.g. PCB153, PCB180 and PCB138) and antiandrogenic (PCB138), and some have dioxin-like potentials (e.g. PCB126). Likewise, for OC pesticides, both estrogenic potentials (e.g. toxaphene, β-hexachlorocyclohexane (HCH), dichlorodiphenyltrichloroethane (DDT) and dichlorodiphenyldichloroethylene (DDE)) and antiandrogenic effects (e.g. DDE) have been reported (64), and α-HCH was shown to antagonize the androgen receptor (AR)-mediated effects of the natural ligand dihydrotestosterone (95). Enantioselective effects of α-HCH were demonstrated by Pavlikova et al. (96) and data suggested an interaction with multiple regulatory events controlling AR activity. Furthermore, additive enhancement of hormone actions has been reported in vitro for xenoestrogen and xenoantiandrogen mixtures (97–99) and in vivo for antiandrogens (100).

Studies in human adrenocortical carcinoma cells (H295R) demonstrated that PCB118, PCB153 and PCB126 decrease protein expression and alter steroidogenesis (101). Exposure to PCB118 increased oestradiol and cortisol secretion, whereas exposure to PCB153 elevated oestradiol secretion. PCB126 was the most potent congener, increasing oestradiol, cortisol and progesterone secretion in exposed H295R cells. The alterations in protein regulation and steroid hormone synthesis suggest that exposure to PCB disturbs several cellular processes, including protein synthesis, stress response and apoptosis.

## Human ex vivo studies on the combined effect of the actual serum legacy POP mixture

In Greenland, district and gender differences were observed for POP exposure biomarkers and biomarkers of the combined effect of extracted lipophilic serum POPs on nuclear receptors (Fig. 4.2). A general inverse relationship was found between higher serum legacy POP concentrations and oestrogen receptor (ER), AR and aryl hydrocarbon receptor (AhR) transactivity. A higher frequency of serum samples with antagonistic ER and AR effects was observed for both sexes on the east (Ittoqqortoormiit, Tasiilaq) and north-west (Qaanaaq) coast of Greenland, whereas higher frequencies of serum samples with agonistic ER and AR effects were observed for both sexes on the west coast (Qeqertarsuaq, Narsaq, Nuuk, Sisimiut). However, for men in Nuuk and Sisimiut, a tendency towards increased serum POP-induced AR activity was observed (Fig. 4.2) (93,94).

Using a specific method for serum extraction of dioxin-like compounds, more than 75% of the serum POP extracts from both sexes elicited AhR-mediated dioxin-like activities. As seen for the hormone receptor transactivities, the tendency was the higher the serum legacy POP levels the lower the AhR transactivities (Fig. 4.2). The lowest medians of the tetrachlorodibenzo-p-dioxin (AhR-TCDD) and toxic equivalence (AhR-TEQ) values were observed in Ittoqqortoormiit (East) and Qaanaaq (North-west), with higher AhR-TEQ levels for both sexes observed in Tasiilaq (only five individuals), Narsaq, Sisimiut and Nuuk, and the highest in Disko Bay (Qeqertarsuaq) (Fig. 4.2) (93,94). A tendency towards an inverse relation between the dioxin-like-induced AhR and ER activity supports the perception that dioxins exert an antiestrogenic effect. Thus, the actual mixtures of serum POPs in Greenlandic Inuit have a hormone-disrupting potential.

Similar data for ER and AhR transactivity were observed using the same extraction method for whole blood from East Greenlandic polar bears. Compared to Inuit, a higher frequency of agonistic xenohormone activity and higher AhR-TEQ levels were found in polar bears which might be explained by higher levels of blood hydroxylated PCBs and higher overall POPs, respectively (102).

In a comparison of Inuit and young Danish women, the POPs levels in Inuit were found more than 10 times higher than in Danes. Moreover, levels were positively associated with age in both study groups. The AhR-TEQ level was significantly higher in Inuit and was positively associated with plasma POPs, whereas no correlations were found for the Danish samples (94,103). Comparisons between European and Greenlandic male serum POP levels showed significantly higher levels in Inuit, and as a result lower ER and AhR transactivity and a tendency towards higher AR activity for the Greenlandic serum samples. However, in the same study, Inuit had significantly lower sperm DNA damage (94).

## Determinants and effects of AhR function

Plasma POP interaction with the AhR signalling pathway was studied by AhR-mediated transcriptional activity in plasma extracts from 874 Inuit adults in Nunavik. Several sociodemographic, anthropometric, dietary and lifestyle variables were considered as possible modulating factors in the AhR-mediated activity in multivariate statistical analyses. The geometric mean AhR-mediated activity expressed as 2,3,7,8-TCDD equivalents was 8.9 µg/kg lipid. PCB153 concentration (Pearson's r=0.53, p<0.001), age and n-3 fatty acids in erythrocyte membranes (p<0.001) correlated positively with AhR-mediated activity, but negatively for body fat mass (p=0.037). The AhR-mediated transcriptional activity was suggested as linked to plasma OC body burden, dioxin-like PCBs, polychlorinated dibenzo-*p*-dioxins and polychlorodibenzofurans (104).

## Perfluoroalkyl acids and endocrine disruption

Ex vivo and in vitro studies have demonstrated endocrine-disrupting potentials of the perfluoroalkyl acids (PFAAs). Estrogenic properties of PFAAs were reported in human Michigan Cancer Foundation-7 breast cancer cells (105), and the endocrine-disrupting potential of seven PFAAs was demonstrated in mammalian cell culture models (106,107). Agonistic effects on ER transactivity were elicited by PFOS, perfluorooctanoic acid (PFOA) and perfluorohexane sulfonate (PFHxS); perfluorooctanesulfonate (PFOS), PFOA, PFHxS, perfluorononanoic acid (PFNA) and perfluorodecanoic acid elicited antagonistic effects on AR transactivity, while the mixture including all seven PFAAs showed additive combined mixture effects. PFDA also weakly decreased the aromatase activity at a high test concentration (106). The seven PFAAs tested also affected thyroid-hormone function by inhibiting rat pituitary growth hormone (GH3) cell growth, and four also antagonized the T3-induced GH3 cell growth (107). Only perfluorododecanoic acid and PFDA elicited an activating effect on AhR transactivation (107). In human serum extracts containing the actual PFAA mixtures, a concentration-dependent agonistic xenoestrogenic activity was found (108). Perfluorinated compounds, and their metabolites present in food packaging materials, were reported to affect steroidogenesis in H295R human adrenal cortico-carcinoma cells (109). A case-control study showed a significant association between PFAA serum level and breast cancer risk (110).

## Contaminant exposure and hormone levels

### Impact on the hypothalamo-pituitary-gonadal axis

A study of reproductive hormones in men from Greenland and three European cohorts (Swedish fishermen, Warsaw Poland, Kharkiv Ukraine) (111) reported significant variation in associations between exposure to PCB153 and *p,p’*-DDE and the outcomes. For the Kharkiv group, statistically significant positive associations were found between levels of both PCB153 and *p,p’*-DDE and SHBG, as well as luteinizing hormone, while for the Greenlandic Inuit men there was a positive association between PCB153 exposure and luteinizing hormone. For the pooled data set, there was a positive association between *p,p’*-DDE and FSH levels (β=1.1 IU/L; 95% CI 1.0–1.1 IU/L), whereas the association between PCB153 and SHBG was of borderline significance. Thus, gonadotropin levels and SHBG seem to be affected by POPs exposure, but in a considerable geographic variation (112). Studying differences between men living in south and north of the Arctic Circle in Norway, Haugen et al. (113) found no geographical differences in either mean levels of PCB153 or sperm parameters. However, mean levels of *p,p’*-DDE were higher in the south than that in the north (p=0.02), as were levels of total and free testosterone, whereas FSH levels were lowest in the south. A strong relationship was observed between PCB153 and SHBG levels. The regional differences observed for *p,p’*-DDE, testosterone and FSH were not reflected in semen quality.

In the Faroe Islands, Grandjean et al. (68) studied the possible endocrine disruption of PCBs and found in boys that higher prenatal PCB exposure was inversely associated with serum luteinizing hormone and testosterone; SHBG level was positively associated with both prenatal and concurrent PCB exposure. The findings suggest that delayed puberty might be due to a PCB-affected central hypothalamo-pituitary mechanism.

### Impact on the hypothalamo-pituitary-thyroid axis

There is substantial evidence that perinatal exposure to PCBs and their hydroxylated metabolites decreases thyroid hormone in the offspring. In man, similar effects have been indicated in several epidemiological studies (114). In a systematic review, Salay and Garabrant (115) evaluated 22 studies to look for a possible association between PCB exposure and circulating thyroid hormones and thyroid-stimulating hormone levels in adults and found that PCBs can interfere with thyroid hormone homeostasis.

Dallaire et al. (116) investigated the potential impact of transplacental exposure to PCBs and HCB on thyroid hormone concentrations in neonates from two remote coastal populations in Canada, Nunavik (n=410) and the Lower North Shore of the St Lawrence River (n=260), and found no association between OC levels and reduction in thyroid hormones in neonates from the two populations. Essential nutrients derived from seafood, for example, iodine, may have prevented the negative effects of OCs on thyroid function during foetal development. Dallaire et al. (117,118) found a positive association between hydroxylated metabolites of PCBs and total tT3 concentrations in pregnant Nunavik women (β=0.57, p=0.02), whereas in cord blood PCB153 concentrations were negatively associated with Thyroxine-binding globulin (TGB) levels (β=−0.26, p=0.01). Maternal pentachlorophenol levels and cord blood fT4 concentrations were inversely related, whereas at 7 months of age no association between exposure and thyroid hormones was observed. In Inuit adults (n=623) from Nunavik, Dallaire et al. (117) found that exposure to several polyhalogenated compounds was associated with modifications of the thyroid parameters, mainly by reducing tT3 and thyroxine-binding globulin circulating concentrations.

Audet-Delage et al. (119) found in Inuit women of reproductive age in Nunavik, Canada, that hydroxylated PCBs, pentachlorophenol and PFOS compete with T4-binding sites on transthyretin (TTR), although the data suggested that circulating levels of TTR-binding compounds were not high enough to affect TTR-mediated thyroid hormone transport. Schell et al. (120) observed in breastfed participants, young adults of the Akwesasne Mohawk Nation, Canada (in contrast to non-breastfeed adults), a significant, positive relationships between anti-thyroid peroxidase antibody levels and all PCB groupings, except non-persistent PCBs, *p,p’*-DDE, HCB and mirex.

Bloom et al. (121) observed significant associations between POPs and thyroid hormones in ageing residents of upper Hudson River communities (age range 55–74 years). Among women, DDT+DDE increased T4 and T3; SPCBs in conjunction with PBDEs elicited increases of T3, and SPCBs in conjunction with DDT+DDE elicited increases of T4. For men, estrogenic PCBs and the sum of estrogenic PCBs in conjunction with DDT+DDE were associated with a T3 decrease. Thus, POPs’ influence on thyroid hormones may have clinical implications in ageing populations.

### Dioxin-like PCBs in relation to bone quality/strength

Results from experimental and population studies suggest that some dioxin-like compounds can alter bone metabolism and increase bone fragility. Bone strength in Inuit appears to be lower than in non-indigenous people. In Cree women of Eastern James Bay (Canada), Paunescu et al. (122) found that for plasma extracts, dioxin-like transactivity, related to PCB105 and PCB118, was inversely associated with the stiffness index. Age, height, smoking status, menopausal status and the percentage of n-6 PUFAs in erythrocyte membranes were negatively associated with one of the ultrasound parameters, while the percentage of n-3 PUFAs in these membranes and levels of physical activity and education were positively associated. Thus, increase in plasma concentrations of PCB105 and PCB118 was negatively and n-3 PUFAs was positively associated with bone stiffness index. In contrast, in Inuit women from Nunavik, neither total plasma dioxin-like compounds nor specific dioxin-like PCBs were associated with stiffness index after adjusting for several confounding and co-varying factors (123).

### POPs and Type 2 diabetes

Several descriptive epidemiology studies suggest that certain POPs can contribute to the development of Type 2 diabetes (124,125). Persistent environmental chemicals still in current use are suspected to be diabetogenic, for example, the brominated flame retardants and perfluorinated compounds. A causal relationship is supported by follow-up of subjects poisoned by PCBs and related substances (126). Most of the recent epidemiological evidence is from cross-sectional case-control studies and found increased serum POP concentrations to be a major determinant of diabetes (127) and metabolic syndrome (128).

Genetic predisposition to Type 2 diabetes seems to play a role, but most genetic variants so far identified are associated with β-cell function and account for no more than about 10% of the risk. Thus, it is very likely that POP exposure may trigger gene–environment interactions with effects on insulin resistance and/or secretion.

Experimental data-based effects of crude fish oil compared to fish oil that had been cleaned of POPs have shown that certain POPs may increase insulin demand by decreasing insulin sensitivity in target tissues (129). In addition, 2,3,7,8- TCDD is known to cause toxicity to pancreatic β-cell lines, such as interference with mitochondrial membrane potential, and induction of increased Ca scientific justification therefore exists for exploring the possible role of POP toxicity in Type 2 diabetes aetiology and pathogenesis.

In the Faroe Islands in 713 septuagenarians with a high POP exposure, a fasting insulin concentration decreased by about 8% for each doubling of the serum concentration of PCBs and a similar increase in the fasting glucose level. Along with higher PCB exposure in subjects with Type 2 diabetes and impaired fasting glycaemia, the results suggest that PCB-induced β-cell deficiency may be involved in the disease pathogenesis (130). Individuals with vitamin D levels <50 nmol/L doubled their risk of newly diagnosed Type 2 diabetes suggesting vitamin D may provide protection against Type 2 diabetes (131).

## Carcinogenic effects

Throughout the 20th century, the cancer patterns of the Inuit population have been characterized by a high risk of Epstein–Barr virus -associated carcinomas of the nasopharynx and salivary glands, and a lower risk of tumours common in Caucasian populations, including cancer of the breast, prostate, testis and hemopoietic system. Both genetic and environmental factors seem to be responsible for this pattern. Over the past 50 years, Inuit societies have undergone major changes in lifestyle and living conditions. The incidence of traditional Inuit cancers (nasopharynx and salivary glands cancer) has remained relatively constant, whereas the incidence of lifestyle-associated cancers, especially cancer of the lung, breast, stomach and colorectal, has increased considerably following changes in lifestyle (smoking, alcohol), diet and reproductive factors (132).

The age-standardized cancer incidence rate for all cancer sites (1998–2007) was found to be 14% lower for the Inuit Nunangat male population and 29% higher for the female population compared to the rest of Canada. Cancers of the nasopharynx, lung and bronchus, colorectal, stomach (males), and kidney and renal pelvis (females) were elevated in the Inuit compared to the rest of Canada, whereas prostate and female breast cancers were lower. Higher smoking prevalence within Inuit Nunangat and distinct socio-economic characteristics between the respective populations may have contributed to the incidence differentials (133).

Some cancer incidence, such as nasopharyngeal, oesophagus, biliary, ventricle, cervical, lung, liver, pancreas and colorectal cancer, in Greenland is several times higher compared to Denmark(The Danish National Patient Register).

The indigenous coastal Chukchi and Inuit living in Chukotka (Russia) are at higher risk of death from cancer during 1961–1990 than the Russian population nationally, among men and women twice and 3.5 times, respectively. Particularly high mortality from oesophageal cancer and lung cancer is seen in the indigenous people of coastal Chukotka. The mortality pattern of incidence corresponds to other indigenous people of the Russian Arctic (134). The incidence of colorectal cancer is currently higher in Alaskan Inuit than in Caucasians living in the United States (132). Cancer is now the leading cause of death among Alaska Native people, and cancer mortality rates in Alaska are significantly higher than in the mainland United States (135).

According to epidemiological studies, about 80% of all cancers are suspected to be related to environmental factors such as contaminant exposure and lifestyle.

## Contaminant exposure, oxidative stress and carcinogenicity

Oxidative stress plays an important role in carcinogenicity (136). Epigenetic mechanisms not involving DNA attack or heritable genetic alterations have been shown to produce tumours in laboratory animals for several chemicals (137). These non-genotoxic carcinogens may target nuclear receptors, cause aberrant DNA methylation at the genomic level and post-translational modifications at the protein level, thereby affecting key regulatory proteins, including oncoproteins and tumour suppressor proteins (138).

Recently, PCBs and polybrominated biphenyls were classified by the International Agency for Research on Cancer as “human carcinogen” and “possible human carcinogen,” respectively (139). Overall, PCBs possess carcinogenicity through inducing formation of reactive oxygen species, genotoxic effects, immune suppression, an inflammatory response, and endocrine effects to various extents and via different pathways. The dioxin-like PCBs exert their effects mainly through AhR activation; less-chlorinated PCBs act more readily through metabolic activation. Mixtures might have more than additive effects. OC pesticides elicit carcinogenicity mainly through non-genotoxic effects. The *o,p’-*DDT, *p,p’*-DDE, and *p,p’*-dichlorodiphenyldichloroethane (*p,p’*-DDD) are able to modulate several cancer-related processes in breast cancer cell lines (140).

Perfluorinated chemicals are suspected carcinogens, and oxidative stress is a possible mechanism of action (141–144). Oxidative stress is also involved in the carcinogenetic effect of PBDEs (145) and heavy metals including arsenic, cadmium, chromium, cobalt, lead, mercury and nickel (146–151). Cadmium can also mimic the in vivo effects of oestrogen in reproductive tissues (152). Thus, cadmium might be related to the development of hormone-dependent cancer such as breast cancer (153).

In blood samples of an Inuit population from Salluit (70 women, 33 men, Canada), the known oxidative lesion, 8-oxodG, DNA adduct was predominant. Some individual adducts appear to accumulate with increasing PCB level, but a definitive association between PCBs and other newly detected DNA adducts could not be made (154). Further investigation of Inuit from Salluit (56 women, 27 men) showed the DNA adduct levels to be inversely associated with the ratio of selenium and PCB levels. In the high Se:PCB ratio group, a significantly negative effect on 8-oxodG (r=−0.38, p=0.014) and total adducts (r=−0.41, p=0.009) was observed, while there was no correlation within the low Se:PCB group (155).

## Contaminant exposure and cancer risk in Arctic regions

### Lung cancer

The incidence of lung cancer has increased remarkably in all Inuit populations over the past 40 years and now constitutes about 20% of all cancers in Inuit (132). The lung cancer incidence in circumpolar Inuit is among the highest in the world, for men and women. The age-standardized incidence rate of lung and bronchus cancer during 1998–2007 of male Inuit from Nunangat was 113 per 100,000 which was double that for the rest of Canada (50.6 per 100,000) (133). Greenland Inuit have double the standardized incidence rate of lung cancer in Denmark (NORDCAN). The smoking pattern among Inuit, possibly combined with co-factors related to environment and diet, is believed to be the relevant causal factors (156). Although modern housing conditions have decreased exposure to fumes from lamps and open fires for cooking, many Inuit still spend substantial periods out on the land, cooking on open stoves inside tents. Marijuana smoking in 85% of adults (of Nunavik, Canada) might also play a role in the high incidence of lung cancer (157).

### Breast cancer

Breast cancer is the most common cancer for women in the western world. The established risk factors include genetic inheritance, for example, mutations in the *BRCA1* and *BRCA2* genes (158), lifelong exposure to oestrogens, obesity after menopause, alcohol, smoking and high fat intake (159). The known risk factors explain less than a third of all cases and more than 70% of women diagnosed with breast cancer have no inherited or sporadic cancer. Risk is thought to be modified by lifestyle and environmental exposure (160). Although still lower in the Arctic Inuit, the frequency is now approaching incidences recorded in Western populations (161) and today about 12 to 15 women are diagnosed every year in Greenland. From 1988 to 1997, the age-adjusted incidence rate for women in Greenland was 46.4 per 100,000. For comparison, the rate in the United States was 124 per 100,000 for 2001 to 2008 and in Denmark about 100 per 100,000 in 2010 (159). The age-adjusted incidence rate for breast cancer in the Arctic Inuit Nunangat was lower than for the rest of Canada (45 vs. 81 per 100,000) (133). A significant increase in breast cancer rate in Alaska Native women was reported during 1974–2003 (162).

The enormous transition in health conditions and lifestyle in the Arctic might be contributing to the known risk factors. PCB exposures have been associated with effects relevant to breast cancer development such as estrogenic tumour promotion (163). Although conflicting data for PFOA exposure in rats and fibroadenomas risk (164), in mice PFOA was associated with altered mammary gland development and differentiation among exposed dams (165). Bonefeld-Jørgensen et al. (110) observed for the very first time a significant association between serum PFC levels and the risk of breast cancer breast cancer risk in Greenlandic Inuit during 2000–2003. The breast cancer cases also had a significantly higher concentration of PCBs at the highest quartile. Also, the combined serum legacy POP-induced agonistic AR transactivity showed an increased odds ratio, and cases elicited a higher frequency of samples with significant POP-related hormone-like agonistic ER transactivity. The AhR toxic equivalent was lowest in breast cancer cases possibly owing to receptor inhibition by non-dioxin-like POPs. The level of serum POPs, particularly PFCs, might be a risk factor in the development of breast cancer in Inuit. Hormone disruption by the combined serum POP-related xenoestrogenic and xenoandrogenic activities may contribute to the risk of developing breast cancer in Inuit (110). Moreover, genetic analyses showed that the *BRCA1* founder mutations and polymorphism in P450 1A1 and CYP17 might increase risk among Greenlandic women (166) A study in southern Quebec found an association between increased levels of PCBs, particularly PCB105, PCB118 and PCB156, and breast cancer (167).

### Ovarian cancer and prostate cancer

The aetiology of ovarian cancer is not fully understood. Previous results support the hypothesis of long-term elevated oestrogen concentrations as etiologically important for this disease (168). For Arctic populations, the age-standard incidence rate of ovarian cancer among Alaska Native women was significantly lower than for US white women (5.2 vs. 10.5 per 100,000) in 1999–2003. No significant change in the rate of developing ovarian cancer was observed for Alaska Natives during 1973–2003 (162).

Germ-line mutations in the tumour suppressor proteins *BRCA1* and *BRCA2* predispose individuals to breast and ovarian cancer. About 10% of all breast and ovarian cancers are dominantly inherited mainly by mutations in the *BRCA1* and *BRCA2* genes. Harboe et al. (169) found three patients out of nine with ovarian cancer (33%), and one out of 10 breast cancer patients (10%) carrying the *BRCA1* mutation in Greenland. Risk of prostate cancer in Inuit is 10–20% of the risk in the respective national white population (161,170). A recent study showed that the age-standard incidence rate for prostate cancer during 1998–2007 was lower in the Inuit Nunangat population than in the rest of Canada (17 vs. 85 per 100,000) (133).

### Pancreatic cancer

A comprehensive meta-analysis has suggested that tobacco smoking, obesity, Type 2 diabetes mellitus and chronic pancreatitis are risk factors for pancreatic cancer. Kirkegaard (171) reported that the age-standardized incidence rate for pancreatic cancer is 138% higher in Greenland Inuit than in Denmark. This could be partly explained by a higher prevalence of smoking and Type 2 diabetes (171).

## Genetic modifiers

### Gene–Environment interactions in relation to cancer risk

Polymorphisms in relation to environmental cancers are those that modify either the exposure dose or the carcinogenic effect of a given exposure. A functional effect of the polymorphism is a prerequisite for a biological effect. Much of the current molecular epidemiological research aims at identifying those functional polymorphisms and their interaction with environmental factors. These differences in risk of cancer are often called “genetic susceptibility.”

### Genetic polymorphisms and contaminants in the Arctic

The indigenous Arctic population is of Asian descent, and their genetic background is different to that of the Caucasian populations. Relatively little is known about the specific genetic polymorphisms in genes involved in the activation and detoxification mechanisms of environmental contaminants in Inuit and their relation to health risk. Ghisari et al. (172) compared the genotype and allele frequencies of the cytochrome P450 CYP1A1 Ile462Val (rs1048943), CYP1B1 Leu432Val (rs1056836) and catechol-O-methyltransferase (COMT) Val158Met (rs4680) in Greenlandic Inuit (n=254) and Europeans (n=262) and found that the genotype and allele frequency distributions of the three genetic polymorphisms differed significantly between the Inuit and Europeans. For Inuit, the genotype distribution was more similar to those reported for Asian populations. A significant difference in serum PCB153 and *p,p’*-DDE levels between Inuit and Europeans was found, and for Inuit associations were also found between POP levels and genotypes for *CYP1A1*, *CYP1B1* and *COMT*. The data provide new information on gene polymorphisms in Greenlandic Inuit that might support evaluation of susceptibility to environmental contaminants (172). Studying polymorphisms in genes involved in xenobiotic metabolism and oestrogen biosynthesis, *CYP1A1*, *CYP1B1*, *COMT* and *CYP17*, *CYP19* and the *BRCA1* founder mutation in relation to breast cancer risk, Ghisari et al. (164) found that the *BRCA1* founder mutation and polymorphisms in *CYP1A1* and *CYP17* can increase breast cancer risk among Inuit women and that risk increases with higher serum levels of PFOS and PFOA. Serum PFAS levels were a consistent risk factor for breast cancer, but inter-individual polymorphic differences might cause variations in sensitivity to the PFAS/POP exposure.

In the INUENDO study population, including proven-fertile men from Greenland, Warsaw (Poland) and Kharkiv (Ukraine), the effect of exposure to POPs on sperm concentration was seen only in men with a short androgen receptor (AR) gene nucleotide CAG sequence repeat. The data were supported in vitro showing that 4,4’-DDE had the most pronounced effect on the AR activity containing 16 CAG repeats, whereas 28 CAG was the most sensitive variant to a mixture of PCB153 and 4,4’-DDE (173). In another INUENDO study, a linear association was found between sperm DNA fragmentation index and CAG and inhibin B and GGN length, respectively, indicating an association with CAG or GGN repeat length and male reproductive function (174).

### Genetics in relation to lifestyle factors in Arctic populations

Nicotine, the psychoactive ingredient in tobacco, is metabolically inactivated by *CYP2A6* to cotinine. *CYP2A6* also activates pro-carcinogenic tobacco-specific nitrosamines (TSNA). Genetic variation in *CYP2A6* is known to alter smoking quantity and lung cancer risk in heavy smokers. In a cross-sectional study of Alaska Native people, cigarette smokers, smokeless tobacco users and *iqmik* (mixture of tobacco and ash) users with lower *CYP2A6* activity had lower urinary total nicotine equivalents and 4-(methylnitrosamino)-1-(3)pyridyl-1-butanol (NNAL) levels (a biomarker of TSNA exposure). Levels of N-nitrosonornicotine (NNN), a TSNA metabolically bioactivated by *CYP2A6*, were higher in smokers with lower *CYP2A6* activities. Light smokers and smokeless tobacco users with lower *CYP2A6* activity reduced their tobacco consumption. Thus, tobacco users with lower *CYP2A6* activity are exposed to lower pro-carcinogen levels (NNAL) and have lower pro-carcinogen bioactivation (higher urinary NNN) being consistent with a lower risk of developing smoking-related cancers. The study demonstrates the importance of *CYP2A6* in the regulation of tobacco consumption behaviours, pro-carcinogen exposure and metabolism (175).

### Genetic variability and hepatitis in the Arctic

Hepatitis B virus (HepB) infection is highly prevalent in circumpolar indigenous peoples. However, the clinical outcome is extremely variable, such that while hepatocellular carcinoma is uncommon in Canadian Inuit, its incidence is slightly higher in Greenlanders than in Danes, and it is especially high in Alaskan Native people infected with HepB genotypes F (HepB/F) and C (HepB/C). The rate, nature and regional susceptibility of HepB genomic mutations among circumpolar indigenous individuals infected by HepB/B6 (Canada), HepB/D (Greenland) and Alaskan Native people, having subsequently developed hepatocellular carcinoma, found mutations associated with severe outcomes predominated in HepB/F. Differing mutational profiles and genetic variability was observed among different HepB genotypes predominating in circumpolar indigenous patients. The persistently high genetic variability with HepB/B6 despite clinical inactivity could be because of the evolution of a host–pathogen balance (176).

### Genetics in relation to HI in the Arctic

In a cross-sectional survey, the genetic causes of hearing impairment (HI) were investigated among the Inuit with a high prevalence. Mutations in the *GJB2* gene have been identified as a frequent cause of HI. *GJB2* encodes the gap junction protein connexin-26 (Cx26), involved in cochlear K+ homeostasis and is important for mechano-sensory sound transduction. Cx26 mutations explain 15–50% of all non-syndromic HI, but apart from that gene, there is a huge genetic heterogeneity with more than 75 loci or genes for autosomal recessive HI identified. The study group comprised 45 East Greenlanders with HI (median age of 35 years; range: 5–76) and 108 East- and 109 West-Greenlanders as controls. In connexin-26 GJB2, the c.35delG allele frequency was 3.3%. Thus the c.35delG GJB2 mutation occurs in Greenland with LF, and the main causes behind the prevalence of HI in this group are chronic otitis, noise traumas and/or unidentified genetic causes (177).

## Epigenetics

Altered programming may result from epigenetic alterations related to environmental contaminant exposure. Epigenetic alterations are now being linked to several important reproductive outcomes, including early pregnancy loss, intrauterine growth restriction, congenital syndromes, preterm birth and pre-eclampsia (178). The molecular processes in epigenetic regulation that influence pregnancy and the possible diseases in adult life need further research.

Rusiecki et al. (179) analysed the relationship between plasma POP concentrations and global DNA methylation (percent 5-methylcytosine) in DNA extracted from blood samples from 70 Greenlandic Inuit and estimated the global DNA methylation via Alu and LINE-1 assays. They found statistically significant inverse correlation between methylcytosine percent and many of the POP concentrations. This first study on environmental exposure to POPs and DNA methylation levels in an Arctic population has shown that global methylation levels are inversely associated with blood plasma levels for several POPs, and further research is required.

Changes in genome methylation with n-3 PUFA intake and the associations between the diabetes- and cardiovascular disease-related traits were studied in a cross-sectional study of 185 Yup'ik Alaska Native individuals and found 27 differentially methylated CpG sites at biologically relevant regions with epigenome-wide significance (p<1×10^−7^): Regions on chromosomes 3 (helicase-like transcription factor), 10 (actin α2 smooth muscle/Fas cell surface death receptor) and 16 (protease serine 36/C16 open reading frame 67). This indicates an association between biologically relevant epigenetic markers and long-term intake of marine-derived n-3 PUFAs (180).

## Genetic predisposition and methylmercury neurotoxicity

Cognitive consequences at school age associated with prenatal MeHg exposure may need to take into account nutritional and sociodemographic cofactors as well as relevant genetic polymorphisms. At low background exposure equivocal associations between MeHg exposure and adverse neuropsychological outcomes were observed in a Bristol cohort. Heterogeneities in several relevant genes suggest possible genetic predisposition to MeHg neurotoxicity (181).

## Effect modifiers

Most environmental research on the effects of chemicals focuses on single exposures. However, exposure to mixtures of chemicals is ubiquitous in real life (182). Certain chemical substances may target the same organ and induce similar effects in an additive or non-additive way (183). Recent studies suggest a synergistic effect of metal mixtures with neuropsychological outcomes (184) or kidney disease (185). However, studies that examine the effects of chemical mixtures remain limited in humans, and even in experimental animal studies (183).

Methylmercury can cause adverse effects on the developing nervous system, however, long-chain n-3 PUFAs in seafood provide beneficial effects on brain development. In the Faroe Islands and NCDS cohort studies, associations between prenatal exposure to MeHg and neurobehavioural deficits at school age were strengthened after fatty acid adjustment (186).

## Conclusions

### Neurobehavioural effects

Effects associated with MeHg exposure have been documented in humans at successively lower exposures, and it is clear that the developing brain is the most vulnerable organ system. Prenatal exposure to MeHg has been associated with clear effects on the developing brain. Cohort studies in the Faroe Islands have demonstrated that children exposed to MeHg in utero exhibit decreased motor function, attention span, verbal abilities, memory, and other mental functions. Follow-up of these children up to the age of 22 years indicates that these deficits, together with deficits in general mental ability, appear to be permanent. Similarly, a study in Nunavik of child development at age 11 years showed that Hg exposure was associated with poorer early processing of visual information, lower estimated IQ, poorer comprehension and perceptual reasoning, poorer memory functions, and increased risk of attention problems and ADHD behaviour. Some of the adverse effects of MeHg on neurodevelopment may be masked by beneficial effects of seafood nutrients. Neurophysiological assessments of brain function also indicate that post-natal exposure up to the teenage years can cause harm. Thus, both pregnant women and children are at increased risk from MeHg exposure. New studies indicate that certain genetic factors may increase vulnerability to MeHg toxicity. Neurophysiological assessments of children from the Faroe Islands and Nunavik have been less clear with regard to the effects of prenatal exposure to PCBs.

### Immunological effects

Certain environmental pollutants can adversely affect the development of the immune system. Young children in Nunavik have had a high incidence of infectious diseases (meningitis, bronchopulmonary infections, and middle ear infections) for many years. Recent studies to investigate the possibility that this could be partly due to maternal transfer of OCs with known immunotoxic properties during breastfeeding show that prenatal exposure to OCs does increase susceptibility to infectious diseases (in particular otitis media). Most experimental evidence points to the role played by the dioxin-like PCB congeners. Immunotoxic effects have also been seen on routine childhood immunizations. Faroese children exhibiting elevated levels of PCBs and especially perfluorinated compounds showed reduced immune response to routine vaccinations. These findings suggest a decreased effect of childhood vaccinations and may indicate a more general immune system deficit. The implications of inadequate antibody production highlight the need to significantly reduce immunotoxicant exposure in Arctic populations, as well as the need for long-term assessments of the health risks associated with exposure to immunotoxic contaminants.

### Reproductive effects

Many Danish and Faroese men have a low level of semen quality compared with men from other European countries, and there are also indications of lower capacity for testosterone production. Studies of semen quality did not show a relationship with PCB153 or *p,p’*-DDE levels in the blood of Greenlanders; however, sperm motility was inversely related to PCB153 concentration in this population.

### Cardiovascular effects

Conflicting results have been reported regarding the impact of prenatal Hg exposure on blood pressure, with 7-year-old Faroese children exhibiting elevated blood pressure and children from Nunavik showing no association between blood pressure and prenatal Hg exposure. However, elevated blood pressure was found to be associated with Hg exposure among adults from the Faroe Islands and Nunavik. Decreased heart rate variability was associated with cord blood Hg concentrations in Faroese children at ages 7 and 14 years but not in 11-year-old children from Nunavik; however, contemporary blood Hg concentrations in these children from Nunavik were associated with decreased overall heart rate variability parameters. This was also the case for adults from Nunavik and James Bay Cree adults.

### Endocrine effects

EDCs can mimic, interfere or block the function of endogenous hormones and so cause adverse developmental, reproductive, neurological, cardiovascular, metabolic and immune effects in humans. The endocrine-disruptive potential of the actual human serum POP mixture is documented. Exposure during early stages of foetal and neonatal development is especially critical and can disrupt the normal pattern of development in later life. Higher prenatal PCB exposure was associated with lower serum concentrations of luteinizing hormone and testosterone in Faroese adolescent boys, while sex hormone-binding globulin was positively associated with both prenatal and concurrent PCB exposures. DDE was highly correlated with PCBs and showed slightly weaker associations with the hormone profile. These findings suggest that delayed puberty with low serum luteinizing hormone concentrations associated with development exposure to non-dioxin-like PCBs may be due to a central hypothalamo-pituitary mechanism.

Exposure to several polyhalogenated compounds has been associated with modifications in thyroid hormone parameters in Inuit adults from Nunavik. An association between POPs levels and thyroid hormones has also been observed in aging residents in upper Hudson River communities. This influence of POPs on thyroid hormones in aging populations may have clinical significance and merits further investigation.

A potential influence of POPs on type 2 diabetes pathogenesis has also been observed among septuagenarian Faroese with a high POPs exposure free of type 2 diabetes and pre-diabetes: The fasting insulin concentration decreased (8%) for each doubling of the serum concentration of PCBs, and a similar increase in the fasting glucose level. Along with higher PCB exposures in persons with Type 2 diabetes and impaired fasting glycaemia, these results suggest that PCB-induced β-cell deficiency may be involved in the disease pathogenesis. Impaired insulin secretion appears to constitute an important part of the type 2 diabetes pathogenesis associated with dietary exposure to lipophilic POPs. In Faroese, a vitamin D status of less than 50 nmol/L doubled the risk of newly diagnosed type 2 diabetes. Thus, vitamin D may provide protection against type 2 diabetes in older persons.

### Carcinogenic effects

During the latter half of the 20th century, cancer incidence increased substantially among all circumpolar Inuit in the Arctic region, especially for the lifestyle-associated lung, breast and colon cancers. Lung cancer now constitutes about 20% of all cancers in Inuit. Overall cancer rates now seem comparable to those of the United States, Canada and Denmark. The recent change in lifestyle and diet and thus environmental contaminant exposure of the Inuit might play a role in this.

### Effect modifiers

Different chemical substances can interact and induce similar effects in an additive, synergistic or non-additive way and may target the same organ. Because most studies concern human exposure to single chemicals rather than chemical mixtures, negative confounding could cause underestimation of those chemicals causing toxicity (e.g. MeHg and PCBs in seafood) and those having benefits (e.g. long-chain n-3 PUFAs in seafood).
